# Cardiometabolic outcomes among schizophrenia patients using antipsychotics: the impact of high weight gain risk vs low weight gain risk treatment

**DOI:** 10.1186/s12888-022-03746-0

**Published:** 2022-02-19

**Authors:** Rezaul Khandker, Farid Chekani, Brendan Limone, Ellen Thiel

**Affiliations:** 1 Merck & Co., Inc., 2000 Galloping Hill Rd, Kenilworth, NJ 07033 USA; 2 IBM Watson Health, Ann Arbor, MI USA

**Keywords:** Schizophrenia, Antipsychotic, Adherence, Cardiometabolic, Dyslipidemia

## Abstract

**Objective:**

Evaluate the prevalence of cardiometabolic conditions among schizophrenia patients before and incidence after initiation of high (HWGR) and low weight gain risk (LWGR) antipsychotic (AP) regimens.

**Methods:**

A retrospective observational cohort study was conducted using administrative claims data from the IBM® MarketScan Commercial and Multi-State Medicaid Databases. Patients with > 1 medical claim with a diagnosis for schizophrenia and newly initiating AP therapy between 1/1/11–6/30/16 were included. Baseline characteristics were assessed in the 12-months before AP initiation; outcomes over 24-months following AP initiation. Patients were characterized by the AP regimen initiated at the index date. Adherence was defined by a medication possession ratio > 0.8 (medication on hand for 80% of follow-up). Multivariate modeling identified predictors of index AP weight gain risk profile and post-index dyslipidemia.

**Results:**

Two thousand seven hundred forty-eight commercially-insured and 8,748 Medicaid patients met the inclusion criteria. A majority of patients initiated on atypical AP and approximately 30% were adherent to their index AP regimen. Within both payers, patients indexing on LWGR AP regimens were more likely to have pre-index diagnoses of cardiometabolic conditions including hypertension, dyslipidemia, and diabetes. Significant predictors of post-index dyslipidemia included AP adherence and pre-index diabetes. Within both payers, odds of initiating HWGR AP regimens were higher among patients with evidence of drug abuse.

**Conclusions:**

There is unmet need for reducing cardiometabolic consequences for patients on AP therapy and this analysis provides evidence that cardiometabolic conditions often develop during early stages of AP therapy. However, this does not appear to be related to the weight gain risk profile of the AP regimen.

**Supplementary Information:**

The online version contains supplementary material available at 10.1186/s12888-022-03746-0.

## Introduction

Schizophrenia is one of the twenty top leading causes of disability across the globe [1]. Schizophrenia is a debilitating, chronic mental illness in which patients exhibit hallucinations, delusions, along with possible grossly disorganized or catatonic behavior and negative symptoms (i.e. lack of motivation) [2]. Current treatment focuses on reducing symptom burden and preventing relapse [3]. These symptoms, paired with commonly occurring comorbid psychiatric conditions, such as substance abuse and anxiety disorders, lead to social and occupational dysfunction.

Antipsychotic medications are the primary pharmacologic treatment for schizophrenia. Antipsychotics reduce the severity of psychotic symptoms and the incidence of relapse in most patients; [3] however, other health conditions and excess mortality still pose a significant burden in this population. Patients with schizophrenia die on average 25 years earlier than the general population, and most of the excess mortality is due to premature cardiovascular deaths related to dyslipidemia [4]. Schizophrenia patients tend to make little use of health care resources despite poor physical health, [5] which may lead to under-detection of preexisting cardiometabolic comorbidities and risk factors. Therefore, it is important to continue to study the associations between antipsychotics and cardiovascular health among schisoprehnia patients.

It is well documented that most antipsychotics cause some amount of weight gain [6]. Clozapine and olanzapine were most likely to cause weight gain, followed closely by risperidone and quetiapine, with aripiprazole and ziprasidone being least likely to lead to significant weight gain [6]. Although antipsychotic medications with high weight gain risk (HWGR) have been identified, direct evidence linking weight gain risk to other cardiometabolic outcomes using real-world data is sparse.

To help address this gap in clinical understanding, the primary objective of this analysis was to evaluate the prevalence of cardiometabolic conditions among schizophrenia patients prior to initiating first-line antipsycotics and the incidence of these conditions following initiation of antipsychotic regimens using real-world data. The type of antipsychotics initiated and whether baseline clinical characteristics are associated with higher likelihood of being prescribed higher or lower weight gain risk medications were analyzed. We also examined whether the weight gain risk profile of the index antipsychotic regimen impacts the incidence of new cardiometabolic conditions.

## Methods

### Sample

This retrospective cohort study used U.S. administrative claims data from the IBM® MarketScan® Commercial Claims and Encounters Database for the period between January 1, 2011 and December 31, 2016 and the MarketScan Medicaid Multi-State Database for the period between January 1, 2011 and June 30, 2016. The Commercial Database includes the claims of patients and their families who are insured through their employers. The Medicaid Multi-State Database includes claims of millions of patients insured by Medicaid from multiple states. Medicaid coverage differs from state to state, and generally includes healthcare coverage for low-income individuals and individuals with disabilities. This research is not considered human subject research as it does not involve interactions with – or interventions among – participants, and only de-identified data was available. All data were de-identified and fully compliant with the Health Insurance Portability and Accountability Act (HIPAA) of 1996, and therefore the study did not require approval or waiver from an institutional review board [7].

### Patient selection criteria

All patients included were adults initiating antipsychotic therapy for schizophrenia. The schizophrenia diagnosis was defined by one or more inpatient or two or more non-diagnostic (i.e. non rule-out) outpatient claims with a diagnosis of schizophrenia based on International Classification of Diseases (ICD) codes (ICD-9-CM 295.0x–295.6x, 295.8x–295.9x; ICD-10-CM F20.xx). Patients were required to have a prescription filled for an antipsychotic medication 6 months prior to, on, or after the first schizophrenia diagnosis. The date of the first pharmacy claim for antipsychotic medication(s) was deemed the index date. Patients were required to be continuously enrolled with medical and prescription drug benefits throughout the 12 months preceding and 24 months following the index date but were not required to meet any minimum number of inpatient or outpatient services throughout the study period.

Patients with claims for any antipsychotic in the 12 months prior to the index date were excluded. This criterion was applied to select patients that were newly initiating AP therapy after at least one year without such therapy. Patients were excluded if they had a claim with a diagnosis for mild cognitive impairment, dementia, or Alzheimer’s disease during baseline, or bipolar disorder, psychosis due to medication condition, personality disorder(s) or pervasive developmental disorder(s) during the follow-up period. Patients with pharmacy claims for clozapine were also excluded, since clozapine is indicated for treatment resistant schizophrenia [8].

Patients were categorized by antipsychotics with HWGR versus those with low weight gain risk (LWGR). Patients were classed as being on a HWGR index treatment regimen if they had one or more claims for olanzapine, chlorpromazine, iloperidone, paliperidone, quetiapine, risperidone, or mesoridazine. Conversely, patients were classified as initiating a LWGR index treatment regimen if they had one or more claims for fluphenazine, haloperidol, perphenazine, thioridazine, thiothixene, aripiprazole, asenapine, lurasidone, ziprasidone, brexpiprazole, trifluoperazine, or cariprazine [6, 9–11] . Patients with claims for both high and low-risk antipsychotic on the index date were classified in the HWGR cohort.

### Demographic and baseline psychiatric characteristics

In both payer cohorts, demographic characteristics were measured as of the index date. Baseline characteristics were measured during the 12-month baseline period and were identified by the presence of at least one medical claim with a diagnosis code for the condition. Conditions of interest included schizophrenia subtype diagnosis, anxiety and mood disorders, and substance abuse disorders (drug abuse, alcohol abuse, other substance abuse/dependency disorder).

### Antipsychotic medications and weight gain risk profiles

Patients were characterized in terms of the first-line antipsychotic initiated at the index date. Index treatment adherence was measured during the 24-month follow-up period using the Medication Possession Ratio (MPR). The MPR was defined as the sum of the days supplied of the index antipsychotic divided by the total number of days in the follow-up period; that is, number of days' supply that a patient should have received had they obtained the medication as prescribed. For patients who received two or more APs (i.e., combination therapy), the MPR was calculated by averaging the days' supply of both medications and dividing by the total number of days in the follow-up period. For long-acting injectable (LAI) medications, the days of clinical benefit derived from dosing schedules, was used in place of days supply. MPR values range from 0 to 1, where a value of 1 corresponds to 100% adherence. We defined adherence as MPR values equal to or greater than 80%, indicating that patients showed continuous medication use for at least 80% of days in the 24-month follow-up period.

### Cardiometabolic conditions

Cardiometabolic characteristics were measured during the 12-month baseline period and the 24-month post-index period were identified by the presence of at least one claim with a diagnosis code for the condition. Additionally, we recorded cardiometabolic-related medications received during the baseline and 24-month follow-up period using National Drug Codes; these medications included antihypertensives, antidiabetic medications and lipid lowering agents.

### Statistical analysis

All analyses were conducted separately by payer group (commercially-insured versus Medicaid) and stratified by index antipsychotic regimen weight gain risk profile (HWGR versus LWGR). Separate analyses were conducted from the onset of the study based on the two insurance groups (i.e., Medicaid and Commercial claims) due to: potential differences in antipsychotic prescribing patterns (e.g., types of medication prescribed and days supply), differences in available demographic characteristics (e.g., race only available in Medicaid, region only available in commercial), and differences in the patient cost-sharing for medications which may lead to different adherence metrics. No differences were expected initially in terms of medication adherence and HWGR association across the two insurance groups. Differences between index antipsychotic regimen weight gain risk profile strata (HWGR vs LWGR) were assessed using chi square tests for categorical variables and t-tests or analysis of variance (ANOVA) for continuous variables.

A logistic regression model was fit to identify predictors of a dyslipidemia diagnosis in the follow-up period. The model was fit among the subset of patients who did not have baseline dyslipidemia. A second logistic regression model was fit to identify predictors of a patient initiating HWGR versus LWGR antipsychotics. For all analyses, p-values less than 0.05 were considered statistically significant.

## Results

### Descriptive statistics

#### Demographic and psychiatric characteristics

A total of 63,467 commercially-insured schizophrenia patients were identified, of whom 2,748 patients were new initiators of antipsychotics and met the additional eligibility criteria. In the Medicaid database, 218,961 schizophrenia patients were identified, of whom 8,748 met the eligibility criteria (Supplemental Figure 1**)**. The mean (SD) age was 38.7 (18.2) years among commercially-insured patients and 39.9 (13.8) years among Medicaid patients (Table 1). Around 70% of patients in both payer groups were categorized as initiating a HWGR regimen. Patients prescribed a HWGR regimen were significantly younger and more likely to be male compared with their LWGR counterparts. The most common psychiatric comorbidities included major depressive disorder, drug abuse, and other substance abuse/dependency disorder.

**Fig. 1:**
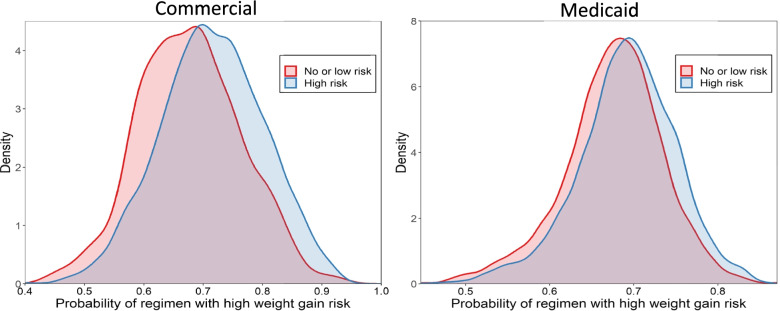
Propensity score for index antipsychotic medication weight gain profile

**Table 1:** Demographic and clinical characteristics at baseline by payer group and index antipsychotic weight gain risk profile

	**Commercial Insurance**			**Medicaid**		
	**All Patients**	**HWGR**	**LWGR**	**All Patients**	**HWGR**	**LWGR**
	***N* = 2,748**	***N* = 1,934**	***N* = 798**	***N* = 8,748**	***N* = 6,004**	***N* = 2,665**
Age (Mean, SD)	38.7 (18.2)	37.3 (18.1)*	41.9 (18.2)*	39.9 (13.8)	39.3 (14.0)*	41.1 (13.1)*
**Gender (N, %)**						
Male	1,627 (59.2%)	1,180 (61.0%)*	442 (55.4%)*	5,067(57.9%)	3,560 (59.3%)*	1,473 (55.3%)*
Female	1,121 (40.8%)	754 (39.0%)*	356 (44.6%)*	3,681 (42.1%)	2,444 (40.7%)*	1,192 (44.7%)*
**Race/ethnicity (N, %)**^**a**^						
White	-	**-**	**-**	2,150 (24.6%)	1,495 (24.9%)*	637 (23.9%)*
Black	-	**-**	**-**	5,402 (61.8%)	3,652 (60.8%)*	1,699 (63.8%)*
American Indian or Alaska Native	-	**-**	**-**	48 (0.5%)	30 (0.5%)*	17 (0.6%)*
Hispanic	-	**-**	**-**	128 (1.5%)	87 (1.4%)*	39 (1.5%)*
Asian or Pacific Islander	-	**-**	**-**	54 (0.6%)	36 (0.6%)*	18 (0.7%)*
Other	-	**-**	**-**	24 (0.3%)	20 (0.3%)*	4 (0.2%)*
Unknown/missing	-	**-**	**-**	942 (10.8%)	684 (11.4%)*	251 (9.4%)*
**Schizophrenia subtype diagnoses (N, %)**						
Paranoid schizophrenia	997 (36.3%)	674 (34.9%)*	316 (39.6%)*	3,657(41.8%)	2,475 (41.2%)*	1,165(43.7%)*
Disorganized schizophrenia	62 (2.3%)	45 2.3%	17 2.1%	315(3.6%)	214(3.6%)	98(3.7%)
Catatonic schizophrenia	45 (1.6%)	34 (1.8%)	9 (1.1%)	74 (0.9%)	54 (0.9%)	20 (0.8%)
Other schizophrenia	1,066 (38.8%)	728 (37.6%)	332 (41.6%)	4,573 (52.3%)	3,153 (52.5%)	1,386 (52.0%)
Schizoaffective disorder	365 (13.3%)	255 (13.2%)	106 (13.3%)	1,516 (17.3%)	1,043 (17.3%)	465(17.5%)
**Other psychiatric conditions (N, %)**						
Generalized anxiety disorder	176 (6.4%)	136 7.0%	40 5.0%	299 3.4%	206 (3.4%)	89(3.3%)
Other anxiety disorder	511 (18.6%)	378 (19.5%)	132 (16.5%)	1,279 (14.6%)	918 (15.3%)*	337 (12.7%)*
Major depressive disorder	589 (21.4%)	413 (21.4%)	172 (21.6%)	1,349 (15.4%)	984 (16.4%)*	355 (13.3%)*
Other mood disorder	317 (11.5%)	229 (11.8%)	83 (10.4%)	884 (10.1%)	639 (10.6%)*	235 (8.8%)*
Drug abuse	451 (16.4%)	364 (18.8%)*	87 (10.9%)*	1,820 (20.8%)	1,331 (22.2%)*	467 (17.5%)*
Alcohol abuse	183 (6.7%)	143 7.4%	38 4.8%	1,099 (12.6%)	778 (13.0%)	308 (11.6%)
Other substance abuse/dependency disorder	338 (12.3%)	247 (12.8%)	90 (11.3%)	2,771 (31.7%)	1,918 (32.0%)	818 (30.7%)
**Cardiometabolic conditions (N, %)**						
Weight gain/obesity	169 (6.2%)	108(5.6%)	60 (7.5%)	1,041 (11.9%)	673 (11.2%)*	350 (13.1%)*
Dyslipidemia	443 (16.1%)	291 (15.1%)*	147 (18.4%)*	1,770 (20.2%)	1,159 (19.3%)*	591 (22.2%)*
Pre-diabetes	66 (2.4%)	47 (2.4%)	19 (2.4%)	260 (3.0%)	175 (2.9%)	83 (3.1%)
Type 2 diabetes mellitus	313 (11.4%)	194 (10.0%)*	114 (14.3%)*	1,386 (15.8%)	859 (14.3%)*	501 (18.8%)*
Hypertension	671 (24.4%)	445 (23.0%)*	220 (27.6%)*	2,978 (34.0%)	1,971 (32.8%)*	960 (36.0%)*
Cardiovascular disease/events	434 (15.8%)	297 (15.4%)	131 (16.4%)	1,192 (13.6%)	818 (13.6%)	351 (13.2%)
Cerebrovascular disease/events	87 (3.2%)	52 (2.7%)*	33 (4.1%)*	310 (3.5%)	209 (3.5%)	92 (3.5%)
Obstructive sleep apnea	102 (3.7%)	74 (3.8%)	27 (3.4%)	230 (2.6%)	140 (2.3%)*	82 (3.1%)*
**Cardiometabolic-related medications (N, %)**						
Antihypertensives	581 (21.1%)	381 (19.7%)*	197 (24.7%)*	2,328 (26.6%)	1,539 (25.6%)*	758 (28.4%)*
Antidiabetic medications	226 (8.2%)	136 (7.0%)*	87 (10.9%)*	969 (11.1%)	584 (9.7%)*	366 (13.7%)*
Lipid-lowering agents	434 (15.8%)	284 (14.7%)*	147 (18.4%)*	1,362 (15.6%)	907 (15.1%)	440 (16.5%)

^*^p < 0.05 between HWGR vs LWGR subgroups

^a^Race/ethnicity data available for Medicaid sample only

*Abbreviations*: *HWGR* High weight gain risk index treatment regimen, *LWGR* Low weight gain risk index treatment regimen, *N* number, *SD* Standard deviation

#### Index antipsychotic medication types, treatment patterns, and adherence

In both payer groups, over 80% of patients were prescribed atypical antipsychotics at baseline. The atypical antipsychotic prescribed the most was risperidone. Around 1 in 5 patients were prescribed a combination of antipsychotics. The mean (SD) number of days supply of the index antipsychotic was 35.1 (23.2) in the commercial sample and 27.4 (8.8) in Medicaid. Less than one-third of both the commercially-insured and Medicaid patients (29% and 31.6%, respectively) were defined as adherent to the index regimen.In the Medicaid sample, a higher proportion of patients initiating a HWGR regimen were adherent compared to those prescribed a LWGR regimen (33.6% vs 27.8, *p* < 0.05). No such differences in adherence were observed in the commercial population (Table 2).

**Table 2:** Antipsychotic medication type, supply, and adherence by payer group and index antipsychotic weight gain risk profile

	**Commercial Insurance**			**Medicaid**		
	**All Patients**	**HWGR**	**LWGR**	**All Patients**	**HWGR**	**LWGR**
	***N* = 2,748**	***N* = 1,934**	***N* = 798**	***N* = 8,748**	***N* = 6,004**	***N* = 2,665**
**Medication type (N, %)**						
Second-generation antipsychotics	2,413 (87.8%)	1,919 (99.2%)*	494 (61.9%)*	7,367 (84.2%)	5,964 (99.3%)*	1,393 (52.3%)*
Lurasidone	47 (1.7%)	16 (0.8%)*	31 (3.9%)*	176 (2.0%)	41 (0.7%)*	135 (5.1%)*
Aripiprazole	485 (17.7%)	140 (7.2%)*	345 (43.2%)*	924 (10.6%)	192 (3.2%)*	732 (27.5%)*
Olanzapine	553 (20.1%)	553 (28.6%)*	0 (0.0%)*	1,209 (13.8%)	1,209 (20.1%)*	0 (0.0%)*
Paliperidone	127 (4.6%)	127 (6.6%)*	0 (0.0%)*	791 (9.0%)	791 (13.2%)*	0 (0.0%)*
Quetiapine	429 (15.6%)	429 (22.2%)*	0 (0.0%)*	1,532 (17.5%)	1,532 (25.5%)*	0 (0.0%)*
Risperidone	1,001 (36.4%)	1,001 (51.8%)*	0 (0.0%)*	2,871 (32.8%)	2,871 (47.8%)*	0 (0.0%)*
Ziprasidone	176 (6.4%)	59 (3.1%)*	117 (14.7%)*	669 (7.7%)	162 (2.7%)*	507(19.0%)*
Other	39 (1.4%)	22 (1.1%)*	17 (2.1%)*	138 (1.6%)	77 (1.3%)*	61 (2.3%)*
First-generation antipsychotics	521 (19.0%)	174 (9.0%)*	331 (41.5%)*	2,142 (24.5%)	652 (10.9%)*	1,421 (53.3%)*
Haloperidol	272 (9.9%)	100 (5.2%)*	172 (21.6%)*	1,451 (16.6%)	433 (7.2%)*	1,018 (38.2%)*
Thiothixene	36 (1.3%)	6 (0.3%)*	30 (3.8%)*	-	-	-
Fluphenazine	94 (3.4%)	24 (1.2%)*	70 (8.8%)*	370 (4.2%)	97 (1.6%)*	273 (10.2%)*
Trifluoperazine	29 (1.1%)	5 (0.3%)*	24 (3.0%)*	-	-	-
Perphenazine	45 (1.6%)	16 (0.8%)*	29 (3.6%)*	109 (1.3%)	29 (0.5%)*	80 (3.0%)*
Chlorpromazine	26 (1.0%)	26 (1.3%)*	0 (0.0%)*	-	-	-
Other	26 (1.0%)	5 (0.3%)*	12 (1.5%)*	199 (2.3%)	111 (1.9%)*	73(2.7%)*
**Route of administration (N, %)**						
Oral	2,605 (94.8%)	1,890 (97.7%)*	705 (88.4%)*	7,615 (87.1%)	5,548 (92.4%)*	2,042 (76.6%)*
LAI	235 (8.6%)	143 (7.4%)*	92 (11.5%)*	1,694 (19.4%)	1,006 (16.8%)*	688 (25.8%)*
Other	76 (2.8%)	40 (2.1%)*	30 (3.8%)*	330 (3.8%)	95 (1.6%)*	181 (6.8%)*
**Medication supply and regimen type**						
Index Antipsychotic Days Supply (Mean, SD)	35.1 (23.2)	33.7 (21.7)*	38.5 (25.7)*	27.4 (8.8)	27.9 (7.8)*	26.8 (10.0)*
Monotherapy	2,192 (79.8%)	1,427 (73.8%)*	749 (93.9%)*	7,158 (81.8%)	4,624 (77.0%)*	2,455 (92.1%)*
Combination therapy	556 (20.2%)	507 (26.2%)*	49 (6.1%)*	1,590 (18.2%)	1,380 (23.0%)*	210 (7.9%)*
**Medication adherence**						
Adherence (*N*, %)^a^	799 (29.1%)	566 (29.3%)	230 (28.8%)	2,763 (31.6%)	2,016 (33.6%)*	740 (27.8%)*
MPR (Mean, SD)	0.46 (0.37)	0.47 (0.37)	046 (0.37)	0.49 (0.37)	0.51 (0.37)*	0.46 (0.36)*

^*^p < 0.05 between HWGR vs LWGR subgroups

^a^Patients with an MPR > 0.80, calculated as the ratio of the number of days’ supply of index treatment to the total number of days in the 24-month post-index treatment date

*Abbreviations*: *HWGR* High weight gain risk index treatment regimen, *LAI* Long-acting injectable, *LWGR* Low weight gain risk index treatment regimen, *MPR* Medication possession ratio, *N* Number, *SD* Standard deviation

#### Pre-index cardiometabolic comorbidities and cardiometabolic-related medications

The most common baseline cardiometabolic comorbidities were dyslipidemia and hypertension. In both payer groups, patients who were prescribed a LWGR regimen were significantly more likely to have underlying type 2 diabetes mellitus compared to patients with a HWGR regimen (Commercial: 10.0% vs 14.3%, *p* < 0.05; Medicaid: 14.3% vs 18.8%, *p* < 0.05). In the Medicaid sample only, patients with a LWGR regimen were also significantly more likely to have diagnosed weight gain/obesity (11.2% vs 13.1%, *p* < 0.05), dyslipidemia (19.3% vs 22.2%, *p* < 0.05), and hypertension (32.8% vs 36.0%, *p* < 0.05) compared to their HWGR counterparts (Table 1).

#### Post-index cardiometabolic comorbidities

Rates of newly incident dyslipidemia were 15.7% and 18.0% in the commercially-insured and Medicaid samples, respectively (Table 3). Similarly, cardiovascular disease or events were diagnosed in 15.8% and 19.5% of the commercially-insured and Medicaid cohorts, respectively. Compared with their HWGR counterparts, LWGR patients in the commercially-insured sample were significantly more likely to have a new diagnosis of weight gain/obesity (9.2% vs 11.9%), type 2 diabetes (4.0% vs 6.7%), or obstructive sleep apnea (2.0 vs 4.8%). Among Medicaid patients, LWGR patients were significantly more likely to have a new diagnosis of weight gain/obesity (13.0% vs 16.0%).

**Table 3:** Post-index cardiometabolic comorbidities by payer group and index antipsychotic weight gain risk profile

	**Commercial Insurance**			**Medicaid**		
	**All Patients**	**HWGR**	**LWGR**	**All Patients**	**HWGR**	**LWGR**
**Cardiometabolic conditions**^**a**^**, N (%)**						
Weight gain/obesity	***N* = 2,579**	***N* = 1,826**	***N* = 738**	***N* = 7,707**	***N* = 5,331**	***N* = 2,315**
	259 (10.0%)	168 (9.2%)*	88 (11.9%)*	1,078 (14.0%)	693 (13.0%)*	371 (16.0%)*
Dyslipidemia	***N* = 2,305**	***N* = 1,643**	***N* = 651**	***N* = 6,978**	***N* = 4,845**	***N* = 2,074**
	363 (15.7%)	251 (15.3%)	109 (16.7%)	1,253 (18.0%)	842 (17.4%)	398 (19.2%)
Pre-diabetes	***N* = 2,682**	***N* = 1,887**	***N* = 779**	***N* = 8,488**	***N* = 5,829**	***N* = 2,582**
	131 (4.9%)	89 (4.7%)	42 (5.4%)	464 (5.5%)	319 (5.5%)	138 (5.3%)
Type 2 diabetes mellitus	***N* = 2,435**	***N* = 1,740**	***N* = 684**	***N* = 7,362**	***N* = 5,145**	***N* = 2,164**
	116 (4.8%)	70 (4.0%)*	46 (6.7%)*	628 (8.5%)	423 (8.2%)	200 (9.2%)
Hypertension	***N* = 2,077**	***N* = 1,489**	***N* = 578**	***N* = 5,770**	***N* = 4,033**	***N* = 1,705**
	300 (14.4%)	210 (14.1%)	89 (15.4%)	1,266 (21.9%)	850 (21.1%)	404 (23.7%)
Cardiovascular disease/events	***N* = 2,314**	***N* = 1,637**	***N* = 667**	***N* = 7,556**	***N* = 5,186**	***N* = 2,314**
	365 (15.8%)	249 (15.2%)	115 (17.2%)	1,477 (19.5%)	1,012 (19.5%)	443 (19.1%)
Cerebrovascular disease/events	***N* = 2,661**	***N* = 1,882**	***N* = 765**	***N* = 8,438**	***N* = 5,795**	***N* = 2,573**
	82 (3.1%)	54 (2.9%)	27 (3.5%)	324 (3.8%)	210 (3.6%)	109 (4.2%)
Obstructive sleep apnea	***N* = 2,646**	***N* = 1,860**	***N* = 771**	***N* = 8,518**	***N* = 5,864**	***N* = 2,583**
	76 (2.9%)	38 (2.0%)*	37 (4.8%)*	254 (3.0%)	157 (2.7%)	89 (3.4%)

^*^p < 0.05 between HWGR vs LWGR subgroups

^a^Conditions developed during the 24-months following index antipsychotic therapy (Denominators depict total number of patients with no evidence of specified diagnosis during 12-months prior to index date)

*Abbreviations*: *HWGR* High weight gain risk index treatment regimen, *LWGR* Low weight gain risk index treatment regimen, *N* number

### Multivariate analyses

#### Post-index dyslipidemia

In both payer groups, medication adherence and type 2 diabetes mellitus at baseline were the strongest predictors of dyslipidemia during the follow-up. In the commercially-insured sample, the odds of dyslipidemia were 1.75 (95% confidence intervals, CI: 1.24–2.47) times higher among patients who adhered to their index antipsychotic regimen and 1.83 (95% CI: 1.25–2.67) times higher among patients with type 2 diabetes mellitus compared with their counterparts. In the Medicaid sample, the corresponding odds ratios were 2.58 (95% CI: 2.13–3.12) for medication adherence and 2.24 (95% CI: 1.85–2.72) for type 2 diabetes mellitus. Baseline cardiovascular disease/events was also a significant predictor (Table 4).

**Table 4:** Multivariate modeling

	**Dyslipidemia During the 24-Month Follow-up Period**				**Index Regimen with High Weight Gain Risk vs Low/No Risk**			
	**Commercial**		**Medicaid**		**Commercial**		**Medicaid**	
**Predictor (baseline characteristics)**	**Odds Ratio**	***P*-value**	**Odds Ratio**	***P*-value**	**Odds Ratio**	***P*-value**	**Odds Ratio**	***P*-value**
Intercept	0.02	< 0.01	0.03	< 0.01	3.52	< 0.01	2.92	< 0.01
Age	1.05	< 0.01	1.04	< 0.01	0.99	< 0.01	0.99	< 0.01
Female versus male	0.63	< 0.01	1.08	0.26	0.92	0.39	0.88	< 0.01
Race: non-white versus white^a^	-	-	0.84	0.03	-	-	-	-
Race: Black vs. White^a^	-	-	-	-	-	-	0.95	0.43
Race: Hispanic vs. White^a^	-	-	-	-	-	-	0.90	0.58
Race: Other/Unknown vs. White^a^	-	-	-	-	-	-	1.14	0.13
Charlson Comorbidity Index	-	-	-	-	0.99	0.89	1.04	0.05
Weight gain/obesity	1.07	0.81	1.43	< 0.01	0.75	0.11	0.88	0.09
Dyslipidemia	-	-	-	-	1.01	0.94	0.92	0.28
Pre-diabetes	-	-	-	-	1.16	0.61	1.07	0.64
Type 2 diabetes mellitus	1.83	< 0.01	2.24	< 0.01	0.93	0.72	0.84	0.08
Hypertension	1.19	0.27	1.46	< 0.01	1.06	0.67	1.00	0.99
Cardiovascular disease/events	1.52	0.02	1.26	0.03	1.04	0.79	1.06	0.46
Cerebrovascular disease/events	-	-	-	-	0.69	0.14	0.91	0.49
Obstructive sleep apnea	-	-	-	-	1.57	0.06	0.77	0.07
Involuntary movements	-	-	-	-	1.27	0.36	0.98	0.87
Seizures/convulsions	-	-	-	-	1.04	0.86	0.86	0.20
Sedation/somnolence	-	-	-	-	1.78	0.06	1.39	0.06
Suicide ideation or behavior	1.36	0.23	1.01	0.95	1.24	0.22	1.00	0.98
Paranoid schizophrenia	0.77	0.05	0.87	0.05	0.87	0.13	0.95	0.31
Disorganized schizophrenia	-	-	-	-	1.23	0.49	0.97	0.81
Other schizophrenia	0.80	0.11	0.81	< 0.01	0.78	0.01	1.03	0.54
Schizoaffective disorder	0.91	0.65	0.96	0.67	0.97	0.81	0.99	0.83
Generalized anxiety disorder	-	-	-	-	1.35	0.13	0.89	0.39
Other anxiety disorder	1.20	0.31	1.14	0.18	1.13	0.31	1.12	0.13
Major depressive disorder	1.10	0.58	1.38	< 0.01	0.82	0.09	1.22	0.01
Bipolar disorder	0.74	0.29	1.31	0.06	1.32	0.13	1.06	0.55
Other mood disorder	1.32	0.19	0.90	0.41	0.87	0.33	1.06	0.49
Conduct disorder	-	-	-	-	1.37	0.18	1.04	0.76
Disruptive behavior disorder	-	-	-	-	1.24	0.33	1.09	0.52
Drug abuse	0.65	0.08	0.71	< 0.01	1.43	< 0.01	1.21	< 0.01
Alcohol abuse	1.11	0.73	0.97	0.81	1.17	0.42	0.99	0.89
Other substance abuse/dependency	0.75	0.20	0.91	0.22	0.97	0.81	0.95	0.35
Delusional disorders	-	-	-	-	1.71	< 0.01	1.16	0.22
SSRI medication	-	-	-	-	0.98	0.87	1.07	0.26
SNRI medication	-	-	-	-	1.01	0.97	0.88	0.33
Anti-anxiety prescription	-	-	-	-	0.91	0.39	0.93	0.26
Anticonvulsant medication	-	-	-	-	0.98	0.86	1.20	< 0.01
Antihypertension medication	-	-	-	-	0.97	0.83	0.95	0.48
Antidiabetic medication	-	-	-	-	0.77	0.23	0.76	0.02
Lipid-lowering medication	-	-	-	-	1.21	0.21	1.21	0.03
HWGR versus low or no risk	1.13	0.40	0.89	0.10	-	-	-	-
MPR	1.75	< 0.01	2.58	< 0.01	-	-	-	-
LAI antipsychotic in index regimen	1.04	0.86	0.83	0.04	-	-	-	-

^a^Race/ethnicity data available for Medicaid sample only

*Abbreviations*: *HWGR* High weight gain risk index treatment regimen, *LAI* Long-acting injectable, *MPR* Medication Possession Ratio, *SNRI* Serotonin and noradrenaline reuptake inhibitor, *SSRI* Selective serotonin reuptake inhibitor

#### Predictors of high weight gain risk vs low weight gain risk regimen

In the Commercial population, the odds of being prescribed a HWGR antipsychotic regimen at index were 1.43 times higher in patients with evidence of drug abuse (95% CI: 1.07–1.91) and 1.71 times higher in patients with delusional disorders (95% CI: 1.25–2.34). In the Medicaid population, the odds of being prescribed a HWGR antipsychotic regimen at index were 1.21 times higher among patients with evidence of drug abuse (95% CI:1.06–1.38) and 1.22 times higher among patients with major depressive disorder (95% CI: 1.06–1.41) (Table 4).

## Discussion

We used real-world administrative claims data to examine the prevalence and incidence of cardiometabolic conditions among schizophrenia patients prior to and following initiation of their antipsychotic regimen. We found high rates of newly incident dyslipidemia, hypertension, and cardiovascular events regardless of payer, suggesting that cardiometabolic side effects are common among patients initiating antipsychotic treatment. Additionally, multivariate regression analyses showed that pre-existing hypertension, type 2 diabetes mellitus, weight gain/obesity and cardiovascular disease/events were significant predictors of developing dyslipidemia. These findings suggest that individuals with preexisting cardiometabolic conditions are at greater risk for dyslipidemia after initiating antipsychotic therapy for schizophrenia. While this finding associating underlying risk factors and the delveopment of dyslipidemia may be intuitive, the analyses futher investigated the role of the types and weight gain risk profiles of antipsychotic medication initiated at index date.

Index antipsychotic weight gain risk profile was not consistently associated with dyslipidemia or related cardiometabolic conditions at follow-up. This finding was somewhat unexpected, as weight gain and obesity, which are expected to be side effects of HWGR medications, have been robustly linked to dyslipidemia and other cardiometabolic conditions. However, it is notable that patients initiating HWGR antipsychotic regimens had lower rates of pre-index cardiometabolic comorbidities. Similar trends were observed in the commercially-insured sample. This finding suggests that pre-existing cardiometabolic conditions and treatments may be a factor influencing provider choice of antipsychotic medication. For example, patients already receiving antidiabetic medications were more likely to initiate on LWGR medications, and we hypothesize that this could be due to physicians channeling patients deemed to be high-risk for other cardiometabolic conditions towards LWGR medications to avoid additional weight gain. This is also consistent with evidence indicating that LWGR antipsychotic regimens are preferred to HWGR antipsychotic regimens in patients who have recently experienced weight gain [12]. In a single-site, proscpective study, Edlinger et al. found that patients’ side effect profiles influenced the physician choice of antipshychotic more than patient demographics or other illness-related variables. Patients who experienced weight gain in the past were less likely to swich to olanzapine (HWGR medication), suggesting that the weight gain risk profile may have been considered in the prescribing decision.

Interestingly, and unexpectedly, these findings did not hold true when modeling predictors of a HWGR profile regimen. Although pre-existing cardiometabolic conditions had no significant impact on the odds of initiating on HWGR vs LWGR regimen among patients in either payer, Medicaid patients receiving anti-diabetic medications did have lower odds of initiating HWGR while those receiving lipid-lowering medication had higher odds. Fig. 1 depicts propensity scores suggesting some minor separation between patients who are predicted to receive HWGR vs LWGR regimens, but there did not appear to be clearly distinct groups. This finding suggests that while some patient channeling is expected to occur in the real-world setting, our analysis did not reveal a true separation by baseline patient characteristics that can completely explain why some patients initiate HWGR and some intiate LWGR.

In contrast to weight gain risk profile, adherence was an important factor contributing to risk for cardiometabolic conditions. Specifically, we found that medication adherence was significantly associated with developing dyslipidemia in both payer groups; this trend may occur because greater medication adherence leads to higher levels of exposure to antipsychotic medications, thereby increasing the risk for adverse side effects. It should be noted, however, that mediation adherence overall was poor, with around two-thirds of patients classified as not adhering to the index antipsychotic regimen. This finding is consistent with previous research [13, 14]. MPR is considered a valid indicator of medication adherence in claims data analyses, as it captures the total days supplied for filled pharmacy claims (i.e. pharmacy claims paid through insurance and not abandoned or not filled by the patient) [14]. The days supplied and relating this number to the total length of the follow-up period is an objective measure of the proportion of days in which the patient had the medication on hand. In this study, no difference was observed in how this adherence measure would perform in either the HWGR group or the LWGR group.

Claims-based studies have several important strengths, including longitudinal follow-up, lack of selection bias, and the availability of standardized data on health care services and filled prescriptions. Nonetheless, this study should be considered in the light of several limitations. First, claims are subject to incomplete, inaccurate, or missing data. In this study, all patients were continuously enrolled in medical and pharmacy benefits for the entire baseline and follow-up periods. Therefore, the data did not miss any claims that were submitted to the patients’ insurance for reimbursement purpose. The analysis was conducted without data imputation considering the possibility of missing information was low. It should again be noted that schizophrenia patients tend to make little use of health care resources despite poor physical health [5]. This phenomena may lead to under-detection of underlying cardiometabolic comorbidities or risk factors, thus, results of this analysis may not be generalizable to the overall schizophrenia population.

A related concern is that pharmacy claims do not indicate whether the medication dispensed was taken as prescribed, potentially overestimating medication adherence. Additionally, the distinction of HWGR vs LWGR antipsychotic medications may be imperfect while research is still being conducted on the newer antipsychotics. Also, so-called “low weight gain” antipsychotics may actually lead to significant weight gain in certain populations. Physicians may be prescribing drugs based on many other considerations and not necessarily considering the weight gain profile of the specific antipsychotic medication.

Second, we cannot establish a causal relationship between antipsychotic initiation and cardiometabolic complications. Although we sought to avoid reciprocal causation by statistically adjusting for pre-index comorbidities, unobserved variables may account for the association between antipsychotic initiation and subsequent cardiometabolic complications, such as genetic predisposition, or unhealthy diet. Our analyses did not specifically investigate dosage-related impacts, where higher dosages may have great impacts on cardiovascular health. Similarly, claims data provide only limited information on factors that influence medication adherence. Patients who have greater insight into their illness and high levels of social support are more likely to adhere to treatment [15]. These factors may also predispose them to seek medical care, which may partly account for the association between medication adherence and being diagnosed with cardiometabolic complications.

## Conclusions

Our findings indicate that there is unmet need for reducing cardiometabolic risk for patients initiating or continuing antipsychotic therapy. Cardiometabolic conditions often develop during the early stages of antipsychotic therapy, and the emergence of these conditions does not appear to be related to the weight gain risk profile of the index antipsychotic regimen. Inconsistencies in our findings across payers may point to unmeasured confounders, such as social determinants of health, influencing incidence of cardiometabolic conditions. While further research regarding these additional patient characteristics is certainly warranted, physicians and patients must also balance the potential cardiometabolic side effects of antipsychotic therapy against control of symptoms and risk for relapse. Finally, all patients with schizophrenia being treated with antipsychotic medications should undergo baseline screening and then regular monitoring of cardiac risk factors.

## Supplementary Information

**Additional file 1: Figure S1** Commercial and Medicaid Sample Selection.

## Data Availability

The data that support the findings of this study are available from IBM Watson Health. IBM is the sole proprietor of the IBM® MarketScan Databases. This data analysis was conducted by IBM researchers under appropriate data use agreements. Restrictions apply to the availability of these data, which were used under license for this study. The datasets used and/or analysed during the current study are available on reasonable request by contacting Ellen Thiel, Researcher, IBM Watson Health—eriehle@us.ibm.com.
