# Supplementary material for: Cardiometabolic outcomes among schizophrenia patients using antipsychotics: the impact of high weight gain risk vs low weight gain risk treatment
Source: BMC Psychiatry. 2022 Feb 19;22:133. doi: 10.1186/s12888-022-03746-0 (PMC8857781; doi:10.1186/s12888-022-03746-0)
Supplement: Supplementary file 1 — Additional file 1: Figure S1 Commercial and Medicaid Sample Selection. [file 12888_2022_3746_MOESM1_ESM.docx]

**Figure 1** Commercial and Medicaid Sample Selection

AND no evidence of a diagnosis for any of the following on or after the index date: bipolar disorder, psychosis due to medical condition, personality disorder(s), or pervasive developmental disorder(s)

**N= 9,884**

Patients in IBM MarketScan® Multi-State Medicaid Database with ≥ 1 non-ruleout inpatient claim or ≥ 2 non-ruleout outpatient claims with a diagnosis of schizophrenia 1/1/2011 – 12/31/2015

**N= 218,961**

AND > 1 claim for an antipsychotic (AP) medication 6 months prior to, on, or after the first schizophrenia diagnosis date; date of earliest AP claim = index date

**N= 133,234**

AND >12 months of continuous enrollment with benefits prior to the index date (pre-index period) and > 24 months following the index date (follow-up period)

**N= 41,859**

AND no evidence of prior AP therapy during the 12 months prior to the index date

**N= 18,939**

AND no evidence of a diagnosis or pharmacy claim for any of the following during the study period: mild cognitive impairment, dementia, Alzheimer's disease, or clozapine use

**N= 8,748**

AND no evidence of a diagnosis for any of the following on or after the index date: bipolar disorder, psychosis due to medical condition, personality disorder(s), or pervasive developmental disorder(s)

**N= 3,275**

Patients in IBM MarketScan® Commercial Database with ≥ 1 non-ruleout inpatient claim or ≥ 2 non-ruleout outpatient claims with a diagnosis of schizophrenia 1/1/2011 - 06/30/2016

**N= 63,467**

AND > 1 claim for an antipsychotic (AP) medication 6 months prior to, on, or after the first schizophrenia diagnosis date; date of earliest AP claim = index date

**N= 42,920**

AND >12 months of continuous enrollment with benefits prior to the index date (pre-index period) and > 24 months following the index date (follow-up period)

**N= 12,580**

AND no evidence of prior AP therapy during the 12 months prior to the index date

**N= 5,946**

AND no evidence of a diagnosis or pharmacy claim for any of the following during the study period: mild cognitive impairment, dementia, Alzheimer's disease, or clozapine use

**N= 2,748**
